# Predictive Value of Clinical and Pathological Characteristics for Metastatic Radioactive Iodine-Refractory Differentiated Thyroid Carcinoma: A 16-year Retrospective Study

**DOI:** 10.3389/fendo.2022.930180

**Published:** 2022-06-28

**Authors:** Jinyan Chai, Ruiguo Zhang, Wei Zheng, Guizhi Zhang, Qiang Jia, Jian Tan, Zhaowei Meng, Renfei Wang

**Affiliations:** ^1^ Tianjin Medical University General Hospital, Tianjin, China; ^2^ Shanghai Tenth People’s Hospital, Tongji University, Shanghai, China

**Keywords:** radioiodine therapy (RIT), radioactive iodine-refractory differentiated thyroid carcinoma, receiver-operating characteristic curve (ROC), thyroglobulin, thyroglobulin antibody (TgAb)

## Abstract

**Purpose:**

To assess predictive value of clinical and pathological characteristics for metastatic radioactive iodine-refractory differentiated thyroid carcinoma (RAIR-DTC) in early stage retrospectively.

**Methods:**

We studied 199 metastatic DTC patients who were divided into two groups (TgAb negative and TgAb positive). The stimulated Tg (Sti-Tg) at the first and second radioiodine therapy (RIT) were defined as Sti-Tg1 and Sti-Tg2, the suppressed Tg (Sup-Tg) were designated as Sup-Tg1 and Sup-Tg2, while the TgAb were defined as TgAb1 and TgAb2, respectively. Univariate analysis and Logistic regression were used to investigate the effects of 13 observed factors to predict RAIR-DTC.

**Results:**

In TgAb negative group, ROC curve analysis showed that cut-off values of age, Sti-Tg2/Sti-Tg1 and Sup-Tg2/Sup-Tg1 to predict RAIR-DTC were 40 years old, 57.0% and 81.0%, respectively. Age, extrathyroid invasion, Sti-Tg2/Sti-Tg1, Sup-Tg2/Sup-Tg1 and BRAF gene mutation were proved to be independent factors predicting RAIR-DTC. In TgAb-positive group, ROC curve analysis showed that cut-off values of age, TgAb1 and TgAb2/TgAb1 to predict RAIR-DTC were 55 years old, 297 IU/ml (14.8 times higher than the upper limit) and 53.6%, respectively.

**Conclusions:**

For TgAb-negative DTC, age over 40, extraglandular invasion, mutated BRAF gene, Sti-Tg decreased less than 43%, and Sup-Tg decreased less than 19% after the first two courses of RIT were independent predictors for RAIR-DTC. For TgAb-positive DTC, age over 55, extraglandular invasion, mutated BRAF gene, distant metastasis before RIT, TgAb level 14.8 times higher than the upper limit, TgAb dropped less than 46.4% after two courses of RIT were influencing factors.

## Background

Thyroid carcinoma is the most common endocrine malignancy, most of which are differentiated thyroid carcinoma (DTC). For decades, “thyroidectomy + radioiodine therapy (RIT) + L-T_4_ suppression therapy” has been considered as standard treatment, the clinical efficacy is well documented (1, 2). However, about 30% of patients with distant metastases become radioactive iodine-refractory DTC (RAIR-DTC) (3, 4), and the 10-year survival rate is less than 10%. Patients would benefit little from RIT if they do not respond or become refractory to ^131^I. Therefore, timely diagnosis of RAIR-DTC can avoid unnecessary RIT and allow patients to move towards other relatively effective treatments. Till now, ^18^F-fluorodeoxyglucose positron emission tomography/computed tomography (^18^FDG-PET/CT) is recommended in clinical guidelines for the identification of RAIR-DTC, combining with ^131^I-whole body scan (^131^I-WBS), computed tomography (CT) and serum thyroglobulin (Tg) level after multiple RIT (3). How to early evaluate, diagnose, and timely adopt effective therapy for metastatic DTC (m-DTC) is a great clinical challenge. This study analyzed the detailed clinical and pathological characteristics of such patients, and proposed a prediction methodology for RAIR-DTC at an early stage.

## Materials and Methods

### Study Conduct

**Figure 1:**
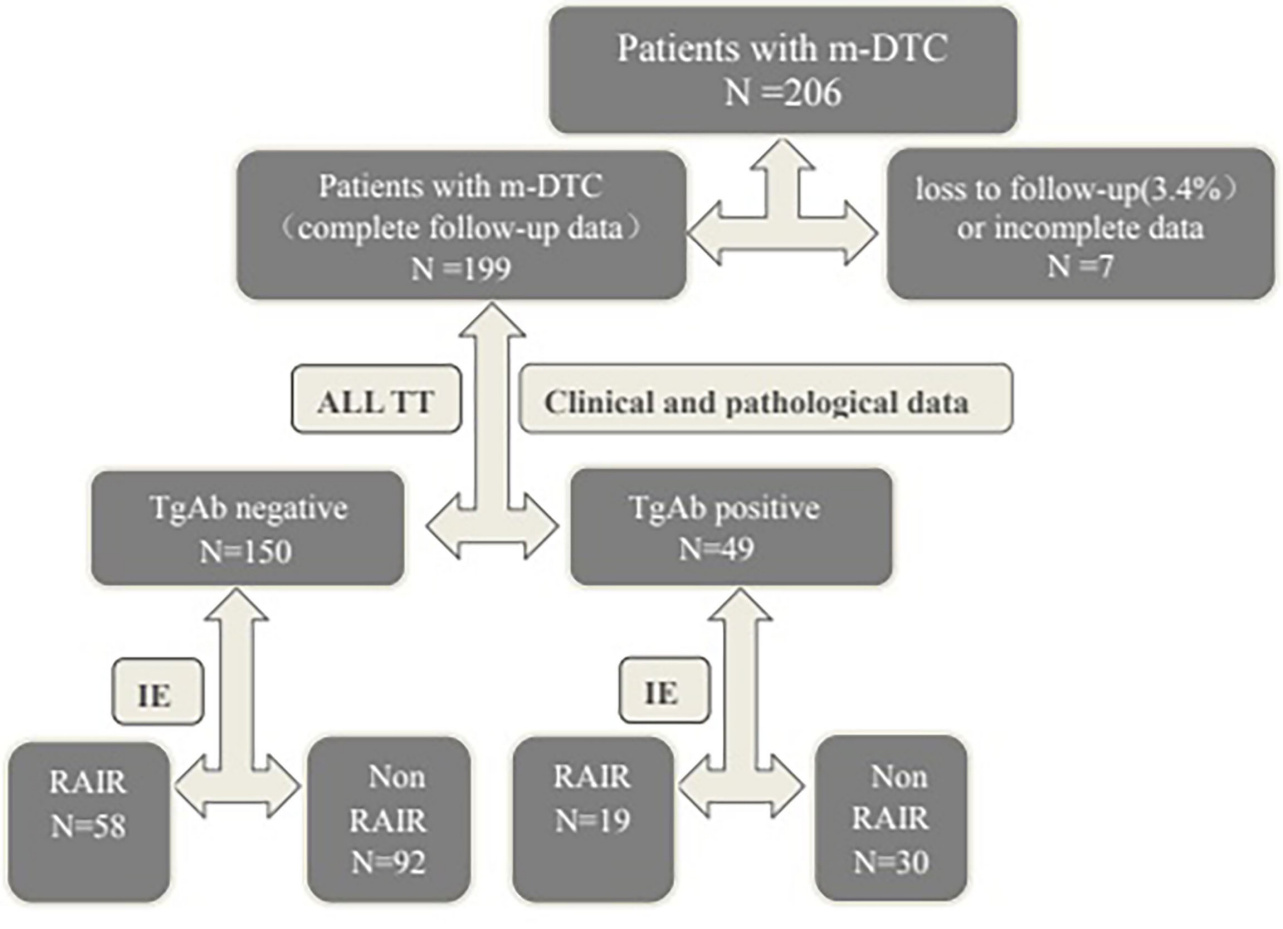
Flowchart of the participants. m-DTC, metastatic differentiated thyroid carcinoma; TT, total thyroidectomy; TgAb, thyroglobulin antibody; IE, imaging examination(WBS, CT, MRI, bone imaging or ^18^F-FDG PET-CT); RAIR, Radioactive iodine-refractory.

Each patient was provided with written information about the principles of the therapy and regulations of radiological protection. Further, written informed consent was obtained from all patients for participating the study. The selection and drop outs of study participants were shown in Flowchart Figure (**Figure 1**).

### Patients

This retrospective study was conducted between January 2003 and October 2019. There were 199 of 206 cases with complete follow-up data. Loss to follow-up rate was 3.4%. Collectively, 199 m-DTC patients (71 males and 128 females; age range: 11-73 years) were enrolled. The average follow-up time was (50.2 ± 20.3) months. The inclusion criteria were as follows: (1) received total or near total thyroidectomy, (2) metastatic lesions existed, (3) received at least two courses of RIT, (4) both Tg and thyroglobulin antibody (TgAb) measured at the same time and scored according to the results. Patients were divided into TgAb negative group and TgAb positive group.

Patients were defined as lung, lymph node, bone or other site of metastasis with the following facts (3): (1) metastasis site confirmed by histopathology, (2) ^131^I-WBS showed focal or diffuse lung, lymph node, bone or other metastatic uptake, with or without other positive findings (by chest CT, X-rays, MRI, bone imaging or ^18^F-FDG PET/CT) and with or without Tg (TgAb) level elevation, (3) m-DTC patients diagnosed with different metastases site by post-therapeutic or diagnostic ^131^I-WBS after thyroid remnant ablation.

### Clinical and Pathological Data

The clinical and histopathological data evaluated in this study included gender, age, primary tumor size, extrathyroid invasion, single or multiple primary tumors, AJCC TNM staging (4), BRAF gene mutation, lung metastasis size, and lymph nodes metastases size. Among them, lung metastasis, and lymph node metastasis were all divided into <1cm and ≥1cm; AJCC T staging was divided into T4 and T3 or below; AJCC N staging was divided into N0 and N1; AJCC M staging was divided into M0 and M1; BRAF gene mutations were detected by PCR.

### Postoperative RIT and Follow-Up Strategy

A serum thyroid stimulating hormone (TSH) level of higher than 30 μIU/mL was achieved by thyroid hormone withdrawal (THW) before RIT. All patients were instructed with a low iodine diet from the beginning of THW till 3 weeks after RIT. Patients were administrated with a radioactive iodine dose of 3.7–7.4GBq (100-200mCi) according to the Chinese guideline (5). The second RIT was performed about 6 months after first therapy (5). All patients were under TSH suppressive therapy by using sodium levothyroxine with TSH <0.1 μIU/mL.

The functional detection sensitivities of Tg and TgAb by electrochemiluminescence immunoassay (Abbott Laboratories, Chicago, IL, USA) were 0.2 ng/mL and 20 IU/mL. TSH was determined by chemiluminescence immunoassay (Abbott Laboratories, Chicago, IL, USA), with a measuring range from 0.35 to 4.94 μIU/mL. A serum stimulated or suppressed Tg, TSH, TgAb were usually followed 1 day before RIT and 2–3 months after RIT.

^131^I-WBS was obtained by using Discovery NM/CT 670 (General Electric Medical Systems, Milwaukee, Wisconsin, USA) equipped with high-energy parallel-hole collimators, and a 20% energy window was centered at 364 keV, at a table speed of 14 cm/min for a total time of 20 min (256×1024 matrix). Post-therapeutic whole-body scan (Rx-WBS) was performed 3-6 days after RIT (5).

### Tg and TgAb Assessment Profiles

The patients were divided into TgAb negative group and TgAb positive group. The stimulated Tg (defined as Sti-Tg) at the first and second RIT were defined as Sti-Tg1 and Sti-Tg2, the suppressed Tg (defined as Sup-Tg) were designated as Sup-Tg1 and Sup-Tg2, while TgAb were defined as TgAb1 and TgAb2, respectively.

### Assessment of Radioactive Iodine-Refractory Thyroid Carcinoma

Till the last time of follow-up, RAIR-DTC was defined if the patient met any of the followings criteria (3): (1) the malignant/metastatic tissue did not ever concentrate RAI, (2) tumor tissue lost the ability to concentrate RAI after previous evidence of RAI-avid disease (absence of ^131^I contamination), (3) RAI was avidly concentrated in some lesions but not in others, and (4) metastatic disease progressed despite avidity of RAI.

### Statistical Analysis

Univariate analysis, t test and Mann-Whitney test were used for qualitative variables, and chi-square test was used to compare categorical data. Logistic regression was used for multivariate analysis. ROC (Receiver operating characteristic) curve analysis was used to determine the critical value for predicting RAIR-DTC. Statistical analysis was carried out using SPSS (version 22.0; SPSS Inc, Chicago, Illinois, USA). A p value of less than 0.05 was considered significant.

## Results

### Patient Clinical and Pathological Characteristics

A total of 199 m-DTC patients (71 males and 128 females; age range: 11-73 years) were involved in this study, 150 were in the TgAb negative group and 49 in the TgAb positive group. Among them, 4 cases were follicular thyroid carcinoma (FTC) and 195 cases were papillary thyroid carcinoma (PTC). A total of 77 patients were considered as RAIR-DTC. The average follow-up time was (50.2 ± 20.3) months. The clinical and pathological characteristics of the included patients were shown in **Table 1**.

**Table 1:** Clinical and pathological characteristics of the included patients.

Characteristics	Case number (%) or value
Gender	
Female	128(64.3)
Male	71(35.7)
Age	
Mean ± SD(years)	45.27 ± 15.23
>55	60(30.2)
≤55	139(69.8)
Tumor size	
Median (IQR) (cm)	1.0(0.8,2.6)
Multifocality/Unifocality	
Multifocality	88(44.2)
Unifocality	111(55.8)
Extra-thyroid extension	
Yes	110(55.3)
No	89(44.7)
AJCC T Stage	
T1	74(37.2)
T2	32(16.1)
T3	36(18.1)
T4	57(28.6)
AJCC N Stage	
N0	46(23.1)
N1a	113(56.8)
N1b	40(20.1)
AJCC M Stage	
M0	98(49.2)
M1	101(50.8)
Before treatment TSH level (μIU/mL)	
First	82.0 ± 37.5
Second	87.4 ± 34.4
RIT dose (mCi)	
First	123.6 ± 29.3
Second	145.3 ± 35.0
Metastasis region	
Pulmonary Only	17(8.5)
Lymph node Only	98(49.2)
Pulmonary and Lymph node	44(22.1)
Pulmonary and Bone	22(11.1)
Pulmonary and other organs	18(9.1)
Lung Node size(cm)	
>1cm	12(6.0)
≤1cm	187(94.0)
Lymph Node size (cm)	
>1cm	11(5.5)
≤1cm	188(94.5)
RAIR	
Yes	77(38.7)
No	122(61.3)
Detection of Non-^131^I-avid Lesions	
CT combined with Tg	69(89.6)
PET combined with Tg	8(10.4)
Follow-up time	
Mean ± SD(month)	50.2 ± 20.3

TSH, thyroid stimulating hormone; RIT, radioiodine therapy; RAIR, radioactive iodine-refractory.

### Univariate Analysis

Univariate analyses on the factors to predict RAIR-DTC were given in **Tables 2**, **3**. The results of TgAb negative group showed that cases with older age, extrathyroidal invasion, higher AJCC T stage, BRAF V600E mutation positivity, lower Sti-Tg2/Sti-Tg1 and lower Sup-Tg2/Sup-Tg1 after the first two courses of RIT would lead to poorer prognosis (P=0.000, 0.000, 0.002, 0.000, 0.000 and 0.000, respectively). However, we found no statistically significant differences in gender, primary tumor size, multifocality, AJCC N stage, AJCC M stage, lung metastasis size and lymph node metastasis size.

**Table 2:** Univariate analysis of the factors in the TgAb negative group.

Baseline factors	Univariate			
	RAIR	non-RAIR	t/Z/X^2^	P
Gender (Male/Female)	24/34	29/63	1.513	0.226
Age	52.190 ± 13.093	37.978 ± 13.937	6.224	<0.001
Tumor Size	1.5(0.9-2.6)	1.0(0.7-1.7)	-1.900	0.057
Extra-thyroid extension (+/-)	55/3	34/58	49.375	<0.001
Multifocality/Unifocality	30/28	40/52	0.9727	0.401
AJCC T Stage(T4/≤T3)	27/31	21/71	9.202	0.002
AJCC N Stage(N1/N0)	50/8	66/26	3.248	0.056
AJCC M Stage(M1/M0)	36/22	51/41	0.643	0.498
Sti-Tg2/Sti-Tg1	1.121 ± 0.484	0.465 ± 0.288	9.337	<0.001
Sup-Tg2/Sup-Tg1	0.967 ± 0.258	0.665 ± 0.397	5.639	<0.001
BRAF (+/-)	47/11	16/76	59.149	<0.001
Lung Node size(>1/≤1cm)	6/52	2/90	4.704	0.056
Lymph Node size(>1/≤1cm)	3/55	3/89	0.339	0.677

**Table 3:** Univariate analysis of the factors in the TgAb positive group.

Baseline factors	Univariate			
	RAIR	non-RAIR	T/Z/X^2^	P
Gender (Male/Female)	4/15	14/16	2.274	0.132
Age	54.105 ± 16.289	42.367 ± 11.559	2.951	0.005
Tumor Size	1.2(0.8-2.5)	0.9(0.7-1.3)	-1.611	0.107
Extra-thyroid extension (+/-)	15/4	6/24	14.186	<0.001
Multifocality/Unifocality	10/9	8/22	3.375	0.066
AJCC T Stage(T4/≤T3)	6/13	3/27	3.613	0.057
AJCC N Stage(N1/N0)	17/2	20/10	2.155	0.142
AJCC M Stage(M1/M0)	12/7	2/28	15.527	<0.001
TgAb1	528.000 (262.000-1652.000)	160.000 (72.750-681.500)	-2.894	0.004
TgAb2/TgAb1	1.046 ± 0.400	0.353 ± 0.199	7.030	<0.001
BRAF (+/-)	11/8	4/26	8.878	0.003
Lung Node size(>1/≤1cm)	2/17	2/28	0.231	0.631
Lymph Node size(>1/≤1cm)	3/16	2/28	0.296	0.587

The results of TgAb positive group showed that cases with older age, extrathyroidal invasion, higher AJCC M stage, higher TgAb1 level, BRAF V600E mutation positivity, and lower TgAb2/TgAb1 would more likely progress to RARI-DTC (P=0.005, 0.000, 0.000, 0.004, 0.003 and 0.000, respectively). However, we found no statistically significant differences in gender, primary tumor size, primary tumor multifocality, AJCC T stage, AJCC N stage, lung metastasis size, and lymph node metastasis size. In specific, cases over 55 years old, with extraglandular invasion, positive BRAF gene mutation, distant metastasis before the first RIT, TgAb level 14.8 times higher than the upper limit, TgAb dropping less than 46.4% after the first two courses of RIT were influencing factors for becoming RAIR DTC.

### ROC Curve Analysis and Diagnostic Performance

Data with statistical significance in the above univariate analyses were further assessed by ROC curve.

**Figure 2:**
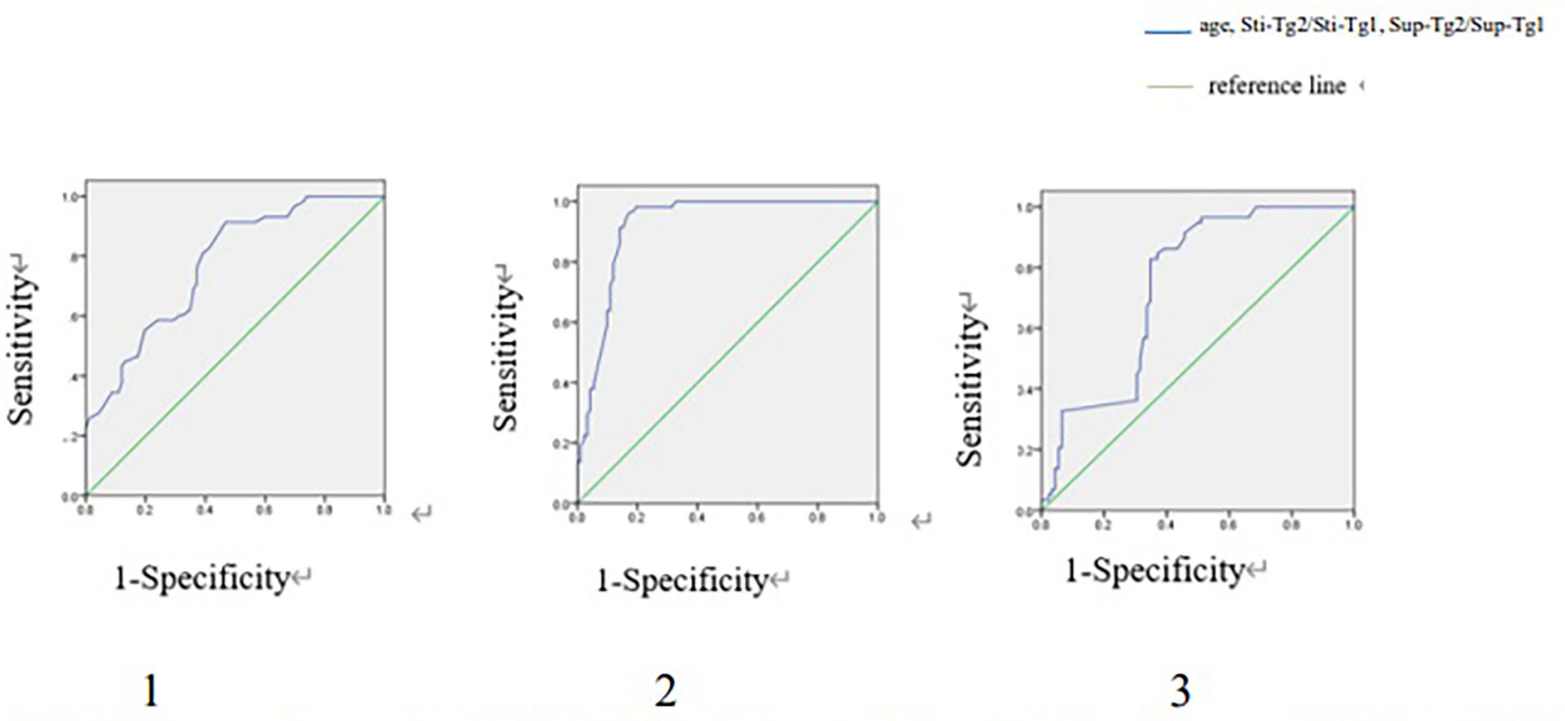
ROC curves of age, Sti-Tg2/Sti-Tg1 and Sup-Tg2/Sup-Tg1 for detecting RAIR in TgAb negative group. ROC, Receiver operating characteristic; RAIR, radioactive iodine refractory. 1. ROC curve of age for detecting RAIR in TgAb negative group. Area under the ROC (AUC) curve 0.772 (95% CI:0.699-0.846), cut-off value 40, sensitivity 0.810, specificity 0.609. 2. ROC curve of Sti-Tg2/Sti-Tg1 for detecting RAIR in TgAb negative group. AUC 0.921 (95% CI:0.878-0.965), cut-off value 0.570, sensitivity 0.983, specificity 0.804. 3. ROC curve of Sup-Tg2/Sup-Tg1 for detecting RAIR in TgAb negative group. AUC 0.740 (95% CI:0.662-0.819), cut-off value 0.810, sensitivity 0.828, specificity 0.652.

In TgAb negative group, ROC curve showed that age, Sti-Tg2/Sti-Tg1 and Sup-Tg2/Sup-Tg1 had good performances in predicting RARI-DTC (**Figure 2**). A cut-off value of age was 40 years old, with a sensitivity of 81.0% and specificity of 60.9% and an area under the curve (AUC) of 0.772. Cut-off value of Sti-Tg2/Sti-Tg1 was 57.0%, with a sensitivity of 98.3% and specificity of 80.4% and AUC of 0.921. Cut-off value of Sup-Tg2/Sup-Tg1 was 81.0%, with a sensitivity of 82.8% and specificity of 65.2% and AUC of 0.740.

**Figure 3:**
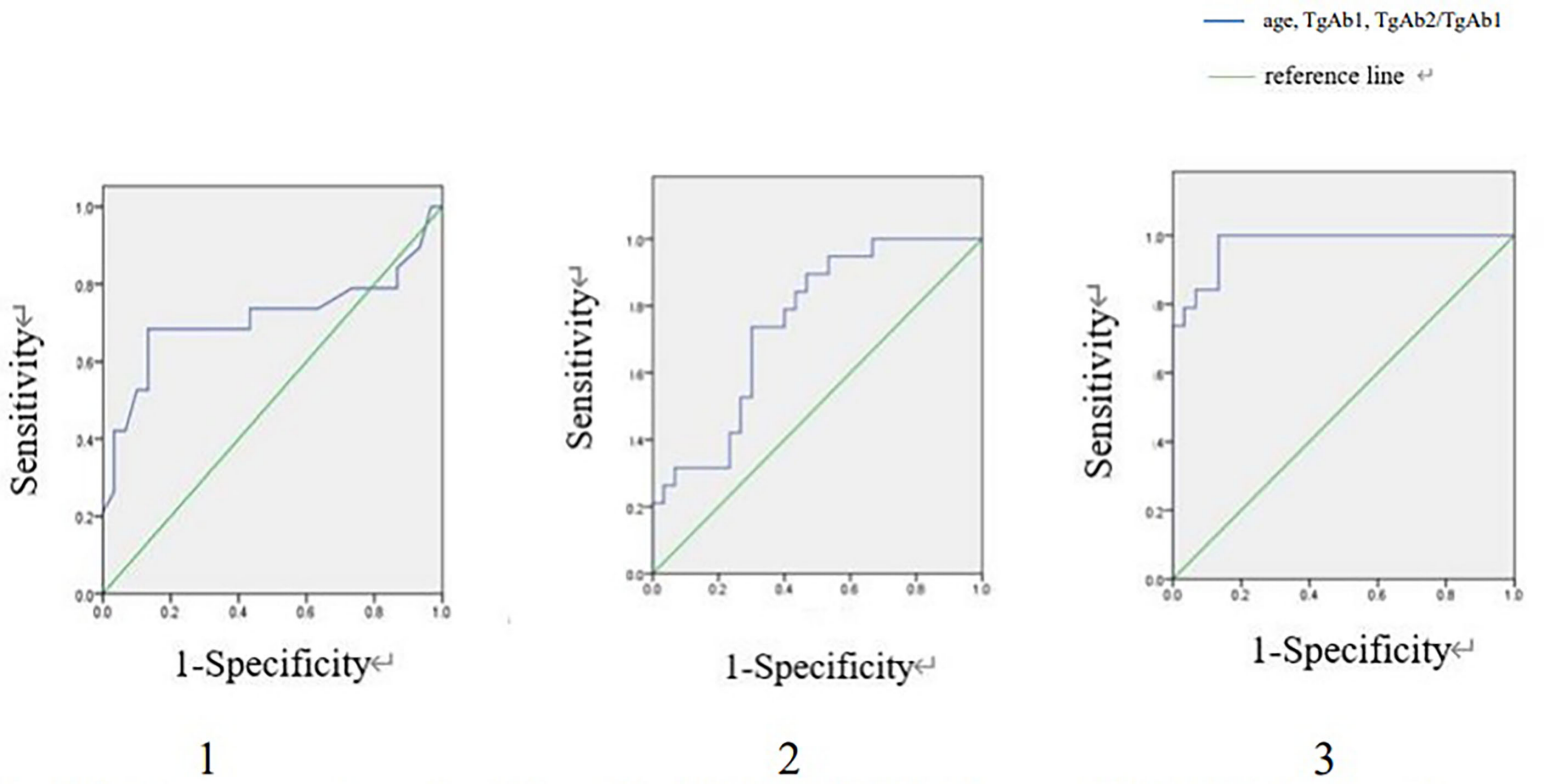
ROC curves of age, TgAb1 and TgAb2/TgAb1 for detecting RAIR in TgAb positive group. ROC, Receiver operating characteristic; RAIR, radioactiveiodine refractory. 1. ROC curve of age for detecting RAIR in TgAb positive group. Area under the ROC curve (AUC) 0.712 (95% CI:0.539-0.885), cut-off value 55, sensitivity 0.684, specificity 0.867. 2. ROC curve of TgAb1 for detecting RAIR in TgAb positive group. AUC 0.747 (95% CI:0.612-0.883), cut-off value:297, sensitivity: 0.737, specificity 0.700. 3. ROC curve of TgAb2/TgAb1 for detecting RAIR in TgAb positive group. AUC 0.974 (95% CI:0.000-1.000), cut-off value 0.536, sensitivity 0.947, specificity 0.867.

In TgAb positive group, ROC curves were drawn regarding age, TgAb1 level and TgAb2/TgAb1 in predicting RARI-DTC (**Figure 3**). The cut-off values of age, TgAb1 level and TgAb2/TgAb1 were 55 years old, 297 IU/ml (14.8 times higher than the upper limit) and 53.6%, with corresponding sensitivities of 68.4%, 73.7% and 94.7%, specificities of 86.7%,70.0% and 86.7%, and AUC values of 0.712, 0.747 and 0.974, respectively.

### Multivariate Logistic Regression Analysis

In TgAb negative group, multivariate logistic regression (**Table 4**) revealed that age (OR: 6.285, 95% CI: 1.256–31.446, P=0.025), extrathyroid invasion (OR: 8.299, 95% CI: 1.521–45.269, P=0.014), Sti-Tg2/Sti-Tg1 (OR: 53.337, 95% CI: 8.630–329.660, P=0.000), Sup-Tg2/Sup-Tg1 (OR: 11.404, 95% CI: 2.270–57.288, P=0.003) and BRAF gene mutation (OR: 7.292, 95% CI: 1.603–33.167, P=0.010) were independent factors in predicting RAIR-DTC. Specifically, age over 40 years old, with extraglandular invasion, BRAF gene mutation positivity, Sti-Tg decreased less than 43% and Sup-Tg decreased less than 19% were independent predictors for RAIR-DTC.

**Table 4:** Logistic regression analyses of factors in the TgAb negative group.

RAIR/non-RAIR	Multivariate			
	P	OR	95%CI	
Age(>40.0/≤40.0)	0.025	6.285	1.256	31.446
Extra-thyroid extension (+/-)	0.014	8.299	1.521	45.269
AJCC T Stage(T4/≤T3)	0.057	6.939	0.948	50.680
Sti-Tg2/Sti-Tg1(>0.570/≤0.570)	<0.001	53.337	8.630	329.660
Sup-Tg2/Sup-Tg1 (>0.810/≤0.810)	0.003	11.404	2.270	57.288
BRAF (+/-)	0.010	7.292	1.603	33.167

Due to small number of cases in TgAb positive group, the logistic regression analysis was not conducted.

## Discussion

Currently, RIT is the mainstay treatment for m-DTC, especially for patients with lung, lymph node, bone or other metastases, while its efficacy depends on the ability of radioiodine uptake in the metastatic lesions. Non-avidity of the lesions usually indicates a tendency towards RAIR and less favorable outcome. The prognosis of patients with m-DTC is affected by many factors, and the loss of iodine uptake capacity in metastases will eventually transform into RAIR-DTC leading to a poorer prognosis (6–8). And the increasing risk of adverse effect and the progression during THW each time from repeated high-dose RIT limited its benefits. The current advancements in RAIR-DTC diagnosis are made according to post-^131^I therapy evaluation, under which circumstance the patients may have been exposed to unnecessary RAI and missed the opportunity to receive more effective interventions (such as targeted drug therapy). There is a need to be able to predict RAIR-DTC before ^131^I therapy and direct to a more individualized treatment (9). How to quickly evaluate and diagnose RAIR-DTC in an early stage, and timely adopt more effective methods for m-DTC is an important issue that needs to be solved urgently (10–12). In the current study, we envision whether metastatic RAIR-DTC can be early predicted by analyzing the clinical and pathological characteristics of these patients.

Multiple studies have confirmed Tg as a specific biomarker of DTC metastasis (13, 14). Song et al. (8) reported that the decline of Tg levels after RIT could link to the efficacy of ^131^I. Serum Tg level is the most sensitive and easily available bio-marker, and an indicator of recurrence or metastasis during the follow-up monitoring after thyroidectomy and ^131^I ablation in DTC patients. The quantitative changes of Tg could provide the basis for tumor response to RIT, and could be used as an index to predict RAIR-DTC (15). Miyauchi et al. (16) found that Tg-doubling time (Tg-DT) was a prominent independent predictor of RAIR-DTC prognosis. Patients with Tg-DT shorter than 1 year showed a 10-year-survival rate of 50%, which was significantly less than 95% of those with Tg-DT for 1-3 years. However, some patients with positive TgAb would hamper the accuracy of Tg determination and makes serum Tg level unreliable. In these patients, Tg cannot be used as DTC marker (17, 18).

In the current management guideline (3), the concept of using TgAb as DTC marker has not been taken into account with a full consensus. There are still conflicts regarding the association between TgAb and DTC outcomes (19–23). Some previous studies suggested that a rising trend of TgAb itself could indicate cancer recurrence or persistence (19–22). However, in another study (23), TgAb was not found to predict disease status in DTC patients. In order to avoid the above inconsistency, this study divided patients into TgAb-negative group and TgAb-positive group for further separate analysis.

Regarding the influencing factors, it is known that age is an independent prognostic factor for the efficacy of RIT (24–26). It could be due to the fact that elderly patients have longer disease duration, lower radiation sensitivity, poor uptake of ^131^I and weaker immune system. Therefore, close follow-up monitoring and/or other management strategies should be carried out for these elderly patients to avoid unnecessary or excessive RIT.

Regarding BRAFV600E mutation in DTC, literature indicates conflicting results. Some viewpoints suggest that BRAFV600E mutation is associated with DTC recurrence and poor prognosis (27–30). The BRAFV600E mutation negatively regulates iodine metabolism genes (NIS, TSHR, Tg, TPO, etc) *via* the abnormal activation of the mitogen-activated protein kinase (MAPK) pathway (31, 32). The opposite viewpoint exists showing BRAFV600E has limited predictive value for DTC prognosis, but, TERT and BRAF double mutations as a biomarker for predicting a worse outcome (33, 34). Our study demonstrated that the positive BRAF gene mutation was a predictor of RAIR-DTC.

In our research, we also focused on whether extrathyroid invasion could lead to RAIR-DTC. Ha’s study suggested that aggressive histopathologic subtypes were significantly associated with the recurrent or metastatic lesions in RAI-negative DTC (35). Robertson et al. (36) conducted a multivariate analysis of 231 patients with thyroid cancer and found that lymphadenopathy was the only important risk factor for recurrence (p<0.001), and the presence of extrathyroid invasion (p=0.013) leaded to high mortality. Aboelnaga (37) found that the survival rate of PTC was significantly affected by age, extrathyroid invasion and extensive distant metastasis. Our study also showed that the presence of extrathyroid invasion could be used as a predictive factor for the diagnosis of RAIR-DTC.

Li et al. (38) evaluated the clinical features of patients with RAIR-DTC, which indicated that tumor size, BMI, gender and TNM staging were statistically nonsignificant trend, however, smoking, tumor type (follicular thyroid cancer), extrathyroid extension, lymph node metastasis number (≥4), lymph node metastasis rate (≥53%), and pN stage (N1) were positively correlated with the prevalence of RAIR-DTC. Our study made some amendments based on the above literature and increased the predictive value of changes in TG and TGAb for RAIR-DTC, and showed negative predictive value for lymph node metastasis size. In the TgAb negative group, those who were over 40 years old, with obvious extraglandular invasion, BRAF gene mutation, Sti-Tg decreased less than 43% and Sup-Tg decreased less than 19% would develop into RAIR-DTC. Even if these patients continue RIT, they would not benefit from it, treatment and follow-up strategy should be adjusted. However, we found no statistically significant differences in gender, primary tumor size, multifocality, AJCC N stage, AJCC M stage, lung metastasis size and lymph node metastasis size. In the TgAb-positive group, the results found that both TgAb levels and trends between RAIR and non-RAIR-DTC were quite different. The persistently high TgAb level may indicate high aggressiveness of the tumor, which was a manifestation of poor prognosis. If the TgAb level did not decrease significantly after the second RIT, the patient would not benefit from the therapy.

This study had limitations. Firstly, the design of retrospective studies has shortcomings and should be verified by future prospective studies. Secondly, the number of TgAb positive cases was small, and it was difficult to conduct multivariate analysis. Thirdly, there may be interactions between positive results obtained by univariate analysis.

## Conclusion

In summary, this study showed for TgAb-negative DTC patients, age over 40, extraglandular invasion, mutated BRAF gene, Sti-Tg decreased less than 43%, and Sup-Tg decreased less than 19% after the first two courses of RIT were independent predictors for RAIR-DTC. For TgAb-positive DTC patients, age over 55, extraglandular invasion, mutated BRAF gene, distant metastasis before the first RIT, level 14.8 times higher than the upper limit, TgAb dropped less than 46.4% after the first two courses of RIT were influencing factors.

## Data Availability Statement

The original contributions presented in the study are included in the article/supplementary material. Further inquiries can be directed to the corresponding authors.

## Ethics Statement

The studies involving human participants were reviewed and approved by Ethical Committee of Tianjin Medical University General Hospital. Written informed consent to participate in this study was provided by the participants’ legal guardian/next of kin.

## Author Contributions

JC and RW contributed to the conception and design of the study. JC, RZ assisted with data acquisition. JC, RZ, and RW conducted the statistical analyses and drafted the manuscript. JT, ZM, QJ and GZ critically revised the manuscript. All authors read and approved the final manuscript and agree to be accountable for all aspects of the research in ensuring that the accuracy or integrity of any part of the work are appropriately investigated and resolved.

## Conflict of Interest

The authors declare that the research was conducted in the absence of any commercial or financial relationships that could be construed as a potential conflict of interest.

## Publisher’s Note

All claims expressed in this article are solely those of the authors and do not necessarily represent those of their affiliated organizations, or those of the publisher, the editors and the reviewers. Any product that may be evaluated in this article, or claim that may be made by its manufacturer, is not guaranteed or endorsed by the publisher.

## References

[1] Donohoe KJ Aloff J Avram AM Bennet KG Giovanella L Greenspan B. Appropriate Use Criteria for Nuclear Medicine in the Evaluation and Treatment of Differentiated Thyroid Cancer. J Nucl Med Off publication Soc Nucl Med (2020) 61):375–96. doi: 10.2967/jnumed.119.240945 32123131

[2] Simsek FS Balci TA Donder Y Ugur K Kilinc F. How Important is the Timing of Radioiodine Ablation in Differentiated Thyroidal Carcinomas: A Referral Centre Experience. Rev espanola medicina Nucl e imagen Mol (2020) 39):157–62. doi: 10.1016/j.remn.2019.08.004 31982352

[3] Haugen BR Alexander EK Bible KC Doherty GM Mandel SJ Nikiforov YE. 2015 American Thyroid Association Management Guidelines for Adult Patients With Thyroid Nodules and Differentiated Thyroid Cancer: The American Thyroid Association Guidelines Task Force on Thyroid Nodules and Differentiated Thyroid Cancer. Thyroid Off J Am Thyroid Assoc (2016) 26):1–133. doi: 10.1089/thy.2015.0020 PMC4739132 26462967

[4] Amin MB Greene FL Edge SB Compton CC Gershenwald JE Brookland RK. The Eighth Edition AJCC Cancer Staging Manual: Continuing to Build a Bridge From a Population-Based to a More "Personalized" Approach to Cancer Staging. CA: Cancer J Clin (2017) 67):93–9. doi: 10.3322/caac.21388 28094848

[5] Tan J Jiang NY Li L Lin YS Lu HK Gao ZR. Guidelines for Radioiodine Therapy of Differentiated Thyroid Cancer (2014 Edition). Chin J Nucl Med Mol Imaging (2014) 34):264–78. doi: 10.3760/cma.j.issn.2095-2848.2014.04.002

[6] Yu L Hong H Han J Leng SX Zhang H Yan X. Comparison of Survival and Risk Factors of Differentiated Thyroid Cancer in the Geriatric Population. Front Oncol (2020) 10):42. doi: 10.3389/fonc.2020.00042 32117715 PMC7008846

[7] Cho SW Choi HS Yeom GJ Lim JA Moon JH Park DJ. Long-Term Prognosis of Differentiated Thyroid Cancer With Lung Metastasis in Korea and its Prognostic Factors. Thyroid Off J Am Thyroid Assoc (2014) 24):277–86. doi: 10.1089/thy.2012.0654 PMC3926138 23758653

[8] Song HJ Qiu ZL Shen CT Wei WJ Luo QY. Pulmonary Metastases in Differentiated Thyroid Cancer: Efficacy of Radioiodine Therapy and Prognostic Factors. Eur J Endocrinol (2015) 173):399–408. doi: 10.1530/eje-15-0296 26104753

[9] Mu ZZ Zhang X Lin YS. Identification of Radioactive Iodine Refractory Differentiated Thyroid Cancer. Chonnam Med J (2019) 55):127–35. doi: 10.4068/cmj.2019.55.3.127 PMC6769251 31598469

[10] Hińcza K Kowalik A Pałyga I Walczyk A Gąsior-Perczak D Mikina E. Does the TT Variant of the rs966423 Polymorphism in DIRC3 Affect the Stage and Clinical Course of Papillary Thyroid Cancer? Cancers (2020) 12):423. doi: 10.3390/cancers12020423 PMC7072153 32059462

[11] Li S Dong S Xu W Jiang Y Li Z. Polymer Nanoformulation of Sorafenib and All-Trans Retinoic Acid for Synergistic Inhibition of Thyroid Cancer. Front Pharmacol (2019) 10):1676. doi: 10.3389/fphar.2019.01676 32116677 PMC7008594

[12] Araque KA Gubbi S Klubo-Gwiezdzinska J. Updates on the Management of Thyroid Cancer. Hormone Metab Res = Hormon- und Stoffwechselforschung = Hormones metabolisme (2020) 52):562–77. doi: 10.1055/a-1089-7870 PMC7415555 32040962

[13] Liu L Zhang X Tian T Huang R Liu B. Prognostic Value of Pre-Ablation Stimulated Thyroglobulin in Children and Adolescents With Differentiated Thyroid Cancer. Thyroid Off J Am Thyroid Assoc (2020) 30):1017–24. doi: 10.1089/thy.2019.0585 31964278

[14] Yang X Liang J Li T Zhao T Lin Y. Preablative Stimulated Thyroglobulin Correlates to New Therapy Response System in Differentiated Thyroid Cancer. J Clin Endocrinol Metab (2016) 101):1307–13. doi: 10.1210/jc.2015-4016 26789779

[15] Wang C Zhang X Li H Li X Lin Y. Quantitative Thyroglobulin Response to Radioactive Iodine Treatment in Predicting Radioactive Iodine-Refractory Thyroid Cancer With Pulmonary Metastasis. PloS One (2017) 12):e0179664. doi: 10.1371/journal.pone.0179664 28704384 PMC5509138

[16] Miyauchi A Kudo T Miya A Kobayashi K Ito Y Takamura Y. Prognostic Impact of Serum Thyroglobulin Doubling-Time Under Thyrotropin Suppression in Patients With Papillary Thyroid Carcinoma Who Underwent Total Thyroidectomy. Thyroid Off J Am Thyroid Assoc (2011) 21):707–16. doi: 10.1089/thy.2010.0355 21649472

[17] Feldt-Rasmussen U Verburg FA Luster M Cupini C Chiovato L Duntas L. Thyroglobulin Autoantibodies as Surrogate Biomarkers in the Management of Patients With Differentiated Thyroid Carcinoma. Curr medicinal Chem (2014) 21):3687–92. doi: 10.2174/0929867321666140826120844 25174917

[18] Spencer CA. Clinical Review: Clinical Utility of Thyroglobulin Antibody (TgAb) Measurements for Patients With Differentiated Thyroid Cancers (DTC). J Clin Endocrinol Metab (2011) 96):3615–27. doi: 10.1210/jc.2011-1740 21917876

[19] Hsieh CJ Wang PW. Sequential Changes of Serum Antithyroglobulin Antibody Levels are a Good Predictor of Disease Activity in Thyroglobulin-Negative Patients With Papillary Thyroid Carcinoma. Thyroid Off J Am Thyroid Assoc (2014) 24):488–93. doi: 10.1089/thy.2012.0611 23971786

[20] Viola D Agate L Molinaro E Bottici V Lorusso L Latrofa F. Lung Recurrence of Papillary Thyroid Cancer Diagnosed With Antithyroglobulin Antibodies After 10 Years From Initial Treatment. Front Endocrinol (2018) 9):590. doi: 10.3389/fendo.2018.00590 PMC6190843 30356857

[21] Qiu ZL Shen CT Sun ZK Song HJ Zhang GQ Luo QY. Lung Metastases From Papillary Thyroid Cancer With Persistently Negative Thyroglobulin and Elevated Thyroglobulin Antibody Levels During Radioactive Iodine Treatment and Follow-Up: Long-Term Outcomes and Prognostic Indicators. Front Endocrinol (2019) 10):903. doi: 10.3389/fendo.2019.00903 PMC6967595 31998236

[22] Sun D Zheng X He X Huang C Jia Q Tan J. Prognostic Value and Dynamics of Antithyroglobulin Antibodies for Differentiated Thyroid Carcinoma. Biomarkers Med (2020) 14):1683–92. doi: 10.2217/bmm-2019-0432 33346697

[23] Smooke-Praw S Ro K Levin O Ituarte PH Harari A Yeh MW. Thyroglobulin Antibody Levels Do Not Predict Disease Status in Papillary Thyroid Cancer. Clin Endocrinol (2014) 81):271–5. doi: 10.1111/cen.12421 24494778

[24] Wierts R Brans B Havekes B Kemerink GJ Halders SG Schaper NN. Dose-Response Relationship in Differentiated Thyroid Cancer Patients Undergoing Radioiodine Treatment Assessed by Means of 124I PET/Ct. J Nucl Med Off publication Soc Nucl Med (2016) 57):1027–32. doi: 10.2967/jnumed.115.168799 26917706

[25] Chen P Feng HJ Ouyang W Wu JQ Wang J Sun YG. Risk Factors for Nonremission and Progression-Free Survival After I-131 Therapy in Patients With Lung Metastasis From Differentiated Thyroid Cancer: A Single-Institute, Retrospective Analysis in Southern China. Endocrine Pract Off J Am Coll Endocrinol Am Assoc Clin Endocrinologists (2016) 22):1048–56. doi: 10.4158/ep151139.Or 27124694

[26] Schneider DF Chen H. New Developments in the Diagnosis and Treatment of Thyroid Cancer. CA: Cancer J Clin (2013) 63):374–94. doi: 10.3322/caac.21195 PMC3800231 23797834

[27] Cho JW Kim WW Lee YM Jeon MJ Kim WG Song DE. Impact of Tumor-Associated Macrophages and BRAF(V600E) Mutation on Clinical Outcomes in Patients With Various Thyroid Cancers. Head Neck (2019) 41):686–91. doi: 10.1002/hed.25469 30659691

[28] Dunn LA Sherman EJ Baxi SS Tchekmedyian V Grewal RK Larson SM. Vemurafenib Redifferentiation of BRAF Mutant, RAI-Refractory Thyroid Cancers. J Clin Endocrinol Metab (2019) 104):1417–28. doi: 10.1210/jc.2018-01478 PMC6435099 30256977

[29] Wang P Lun Y Fu Y Wang F Zhao S Wang Y. Generation of a Potential Prognostic Matrix for Papillary Thyroid Cancer That Assesses Age, Tumor Size, Transforming Growth Factor-β, and BRAFV600E Mutation. Oncol Res Treat (2017) 40):586–92. doi: 10.1159/000477909 28892804

[30] Xing M Alzahrani AS Carson KA Shong YK Kim TY Viola D. Association Between BRAF V600E Mutation and Recurrence of Papillary Thyroid Cancer. J Clin Oncol Off J Am Soc Clin Oncol (2015) 33):42–50. doi: 10.1200/jco.2014.56.8253 PMC4268252 25332244

[31] Xing M. Molecular Pathogenesis and Mechanisms of Thyroid Cancer. Nat Rev Cancer (2013) 13):184–99. doi: 10.1038/nrc3431 PMC3791171 23429735

[32] Yan C Huang M Li X Wang T Ling R. Relationship Between BRAF V600E and Clinical Features in Papillary Thyroid Carcinoma. Endocrine connections (2019) 8):988–96. doi: 10.1530/ec-19-0246 PMC6652244 31252408

[33] Luo Y Jiang H Xu W Wang X Ma B Liao T. Clinical, Pathological, and Molecular Characteristics Correlating to the Occurrence of Radioiodine Refractory Differentiated Thyroid Carcinoma: A Systematic Review and Meta-Analysis. Front Oncol (2020) 10):549882. doi: 10.3389/fonc.2020.549882 33117686 PMC7561400

[34] Meng Z Matsuse M Saenko V Yamashita S Ren P Zheng X. TERT Promoter Mutation in Primary Papillary Thyroid Carcinoma Lesions Predicts Absent or Lower (131) I Uptake in Metastases. IUBMB Life (2019) 71):1030–40. doi: 10.1002/iub.2056 31026111

[35] Ha LN Iravani A Nhung NT Hanh NTM Hutomo F Son MH. Relationship Between Clinicopathologic Factors and FDG Avidity in Radioiodine-Negative Recurrent or Metastatic Differentiated Thyroid Carcinoma. Cancer Imaging Off Publ Int Cancer Imaging Soc (2021) 21):8. doi: 10.1186/s40644-020-00378-z PMC7792294 33413689

[36] Robertson B Parker M Shepherd L Panieri E Cairncross L Malherbe F. Nodal Disease Predicts Recurrence Whereas Other Traditional Factors Affect Survival in a Cohort of South African Patients With Differentiated Thyroid Carcinoma. Cancers Head Neck (2018) 3):10. doi: 10.1186/s41199-018-0037-5 31093363 PMC6460678

[37] Aboelnaga EM Ahmed RA. Difference Between Papillary and Follicular Thyroid Carcinoma Outcomes: An Experience From Egyptian Institution. Cancer Biol Med (2015) 12):53–9. doi: 10.7497/j.issn.2095-3941.2015.0005 PMC4383844 25859412

[38] Li G Lei J Song L Jiang K Wei T Li Z. Radioiodine Refractoriness Score: A Multivariable Prediction Model for Postoperative Radioiodine-Refractory Differentiated Thyroid Carcinomas. Cancer Med (2018) 7):5448–56. doi: 10.1002/cam4.1794 PMC6246937 30264548

